# Systematic Differences between Cochrane and Non-Cochrane Meta-Analyses on the Same Topic: A Matched Pair Analysis

**DOI:** 10.1371/journal.pone.0144980

**Published:** 2015-12-15

**Authors:** Johanna Useem, Alana Brennan, Michael LaValley, Michelle Vickery, Omid Ameli, Nichole Reinen, Christopher J. Gill

**Affiliations:** 1 Department of Epidemiology, Boston University School of Public Health, Boston, Massachusetts, United States of America; 2 Center for Global Health & Development, Boston University School of Public Health, Boston, Massachusetts, United States of America; 3 Health Economics and Epidemiology Research Office, Department of Internal Medicine, School of Clinical Medicine, Faculty of Health Sciences, University of the Witwatersrand, Johannesburg, South Africa; 4 Department of Biostatistics, Boston University School of Public Health, Boston, Massachusetts, United States of America; 5 Department of Global Health, Boston University School of Public Health, Boston, Massachusetts, United States of America; University of Illinois-Chicago, UNITED STATES

## Abstract

**Background:**

Meta-analyses conducted via the Cochrane Collaboration adhere to strict methodological and reporting standards aiming to minimize bias, maximize transparency/reproducibility, and improve the accuracy of summarized data. Whether this results in differences in the results reported by meta-analyses on the same topic conducted outside the Cochrane Collaboration is an open question.

**Methods:**

We conducted a matched-pair analysis with individual meta-analyses as the unit of analysis, comparing Cochrane and non-Cochrane reviews. Using meta-analyses from the cardiovascular literature, we identified pairs that matched on intervention and outcome. The pairs were contrasted in terms of how frequently results disagreed between the Cochrane and non-Cochrane reviews, whether effect sizes and statistical precision differed systematically, and how these differences related to the frequency of secondary citations of those reviews.

**Results:**

Our search yielded 40 matched pairs of reviews. The two sets were similar in terms of which was first to publication, how many studies were included, and average sample sizes. The paired reviews included a total of 344 individual clinical trials: 111 (32.3%) studies were included only in a Cochrane review, 104 (30.2%) only in a non-Cochrane review, and 129 (37.5%) in both. Stated another way, 62.5% of studies were only included in one or the other meta-analytic literature. Overall, 37.5% of pairs had discrepant results. The most common involved shifts in the width of 95% confidence intervals that would yield a different statistical interpretation of the significance of results (7 pairs). Additionally, 20% differed in the direction of the summary effect size (5 pairs) or reported greater than a 2-fold difference in its magnitude (3 pairs). Non-Cochrane reviews reported significantly higher effect sizes (P< 0.001) and lower precision (P<0.001) than matched Cochrane reviews. Reviews reporting an effect size at least 2-fold greater than their matched pair were cited more frequently.

**Conclusion:**

Though results between topic-matched Cochrane and non-Cochrane reviews were quite similar, discrepant results were frequent, and the overlap of included studies was surprisingly low. Non-Cochrane reviews report larger effect sizes with lower precision than Cochrane reviews, indicating systematic differences, likely reflective of methodology, between the two types of reviews that could generate different interpretations of the interventions under question.

## Introduction

In 1972, Archie Cochrane expressed the need for higher quality empirical evidence around the development of health services.[1] Cochrane believed that randomized controlled trials played a major role in the development of this evidence, but realized that there was no systematic way to disseminate results from randomized trials to the professional medical field.[2] As a result, in 1993, The Cochrane Collaboration was established to conduct meta-analytical reviews on health care related topics, specifically randomized trials, enabling physicians and other key decision-makers to access high-quality information on evidence-based results. Because of its rigorous and analytic methodology, standardization of approaches, and transparency, the Cochrane Collaboration is often considered to be the gold standard for meta-analytic reviews, is deemed robust against bias,[3] and is highly trusted by clinicians.[4]

Not infrequently, two or more meta-analyses are independently published on the same topic, though such studies often fail to reference each other’s findings and may yield conflicting results.[5,6] Reviews conducted within the Cochrane Collaboration follow a standardized set of methods that non-Cochrane reviews are not bound to. In theory, this might introduce systematic differences between the two. Several studies provide empirical evidence that Cochrane reviews tend to be of higher quality, were less vulnerable to bias, acknowledged more limitations, and were generally more conservative in how the results were endorsed than non-Cochrane reviews.[7,8] Whether such methodological differences yield different results is an open question.

To contrast and assess the degree of concordance between Cochrane vs. non-Cochrane reviews we conducted a matched pair analysis, comparing pairs of meta-analyses from the Cochrane and non-Cochrane literatures that had examined the same set of interventions and outcomes. Our analysis had four main objectives. First, we wished to contrast the meta-analyses from the two literatures in terms of sample size, numbers of included subjects, date of publication, and the degree to which the studies included in each member of the pair overlapped. Second, we wished to characterize the frequency with which the two literatures conflicted with each other, in terms of significant differences in the magnitude of effect sizes, shifts in the confidence intervals that would lead to differences in a reader’s interpretation of the results. Third, we quantified the degree to which the two literatures differed in terms of summary effect size and statistical precision. Lastly, we assessed the relationship between how frequently meta-analyses were cited as a function of whether and how the results between each matched pair differed.

## Methods

### Overview

Our analysis compared Cochrane and non-Cochrane meta-analyses that reported on interventional randomized controlled trials within the cardiovascular literature. Our selection of the cardiovascular literature was to some degree arbitrary, but was influenced by several considerations. First, it has one of the largest collections of meta-analyses, which we surmised could make it easier to find matches. Second, it is rich in dichotomous outcomes (e.g. myocardial infarction vs. no myocardial infarction, stroke vs. no stroke, death vs. no death, etc.), which was helpful given our intention only to focus on dichotomous outcomes and not continuous variables. Third, it is enriched by a sizeable number of large and methodologically rigorous source studies focused on well-defined interventions around a relatively narrow range of medical outcomes.

### Search, Inclusion and Matching Strategies

To identify studies for the matched analysis, we employed a search strategy in PubMed using the following search terms: [("Cochrane database of systematic reviews (Online)"[Journal] AND (meta analysis [Publication Type]) OR meta-analysis[Publication Type] AND ((((((cardiovascular[MeSH Terms]) OR cardiovascular disease$[MeSH Terms]) OR cardiology[MeSH Terms]) OR heart disease$[MeSH Terms]) OR coronary heart disease$[MeSH Terms]) OR atherosclerosis[MeSH Terms]) OR coronary artery disease$[MeSH Terms]]. The search was executed on 27 October 2012 and included Cochrane and non-Cochrane reviews.

Meta-analyses from the cardiovascular literature were included if they were comprised of 1) randomized controlled trials; 2) conducted on human subjects ≥16 years of age; 3) reported a dichotomous outcome (for ease of working with measures on a risk ratio/odds ratio scale); 4) reported a common treatment-outcome relationship, and 5) were published after 1996, the year that the first Cochrane reviews were published, and the year in which the Cochrane Collaboration’s methodology was first posted to the World Wide Web.[9] In some cases, we identified reviews that updated a previously published meta-analysis. In such cases, we only considered the most recent iteration of that review.

After all inclusion criteria were met, we attempted to match each non-Cochrane meta-analysis with a comparable Cochrane meta-analysis based on:

1. Disease condition;
2. Intervention;
3. Clinical outcome measured; and
4. Publication within 5 years of each other.

We employed a two-step matching process.

First we had to identify pairs of reviews that focused on the same intervention/outcome combinations that met inclusion criteria. Because the number of Cochrane reviews is small compared with non-Cochrane reviews, we found it most efficient to start the matching process within the Cochrane review, and then seek potential non-Cochrane matches.

Second, we had to identify identical analyses within each review pair that described the same intervention and outcomes. Since a given review often includes multiple meta-analyses addressing several endpoints—and in the case of Cochrane reviews, sometimes dozens of endpoints—we used the following approach to identify matches on specific meta-analyses within each pair. Because non-Cochrane reviews tend to report fewer outcomes, we started with the non-Cochrane outcomes and then attempted to match within Cochrane. If the non-Cochrane meta-analysis had a defined primary endpoint, we used that to match into the Cochrane review. If more than one primary clinical endpoint was defined, we used a random number generator to select one, and then attempted to match that to the non-Cochrane paper. If that failed to yield a match, we moved to the next randomly selected endpoint from the non-Cochrane paper to seek a Cochrane match, and so forth until a matched anlaysis was made or all attempts were exhausted.

To avoid putting too much weight on publications that listed multiple potential matches, we only used a single intervention/outcome combination for each pair of meta-analyses, after which we moved on to the next matched pair. More simply stated, each member of each pair in this analysis could only enter our final analytic data set once. This means that our matched pair analysis represents only a small subset of potential matches between the Cochrane and non-Cochrane literatures.

Once we had assembled our set of potential matched pairs from the two sets of reviews, the entire team reviewed the matches to confirm that each pair met our matching criteria, that the extracted data were correct, and that the direction of the effect size was harmonized (which could be violated if one analysis had expressed effect size in terms of a protective effect in the presence of the intervention, whereas the other defined it as harmful effect in the absence of the intervention). In the few instances when this occurred, we re-calculated the effect sizes so that all interventions were expressed as a hypothetical risk reduction due to the intervention.

### Data Extraction

Given the exploratory nature of this analysis, we had no sample size estimates to guide our search. Arbitrarily, we aimed to capture 50 matched pairs (or 100 meta-analyses in total), although in the end we only located 40 matched pairs for the final analysis. The following data were extracted from each meta-analysis: author, year of publication, disease condition, intervention (treatment), comparison (control), cardiovascular outcome, combined effect estimate, 95% confidence interval, sample size and number of studies included. We made no distinction between reviews that reported using odds ratios vs. relative risks, and instead took these as reported in the review. We debated whether to recalculate all results using a common statistic, but ultimately decided against this, reasoning that the average consumer/reader of a meta-analysis would be unlikely to recalculate a relative risk as an odds ratio to see if that might harmonize discrepant results between two reviews. This reasoning also applied to whether one analysis used a different statistical approach, such as fixed vs. random effects modeling. The average reader may or may not appreciate the distinctions between these approaches, but in any case would be exceedingly unlikely to replicate the analyses themselves around a unified statistical model. Thus, we opted to use the data as reported in the papers, just as a typical reader would see the results.

### Statistical Analyses

Descriptive statistics were used to summarize characteristics of each matched Cochrane and non-Cochrane pair. We calculated differences in sample sizes, the number of studies included, and the year of publication between each matched pair. The Wilcoxon two-sample test was used to determine if there was a significant difference in the total number of studies and sample sizes between Cochrane and non-Cochrane reviews. To compare the summary measures of effects within each matched pair, we displayed Cochrane and non-Cochrane summary estimates and corresponding 95% confidence intervals using Forest plots generated via a macro on Microsoft Excel.

We identified pairs with discrepant results, and sorted them based on the nature of the discrepancy using the following categories:

1. Changes of the width of 95% confidence intervals that shifts a statistical interpretation of the meta-analytic result, e.g., one review concludes a statistically significant result and the other non-significant result.
2. The magnitude of the aggregate effect sizes differed by at least 2-fold (but were in the same direction).
3. The direction of the effect size was reversed.

To determine if the summary measures of effect differed significantly between Cochrane and non-Cochrane, we regressed the Cochrane estimate on the matched non-Cochrane estimate on a logarithmic scale using simple linear regression and displayed the results graphically. We repeated the regression analysis using the standard error (SE) from each member of the pair to contrast how precision differed between the two review types.

While somewhat controversial, bibliometric measures such as citation rates are widely used as a proxy for the impact of that paper in the scientific literature.[10,11] To probe the relationship between citation frequencies and discrepant results we used Google Scholar’s search engine, within each category of discordancy and grouped by Cochrane and non-Cochrane to identify the number of times a given review was cited by other studies in the literature subsequently, and displayed these graphically as box/whisker plots. All statistical analyses used SAS version 9.2. The final data sets can be accessed on line at S1 Data.

## Results

**Fig 1:**
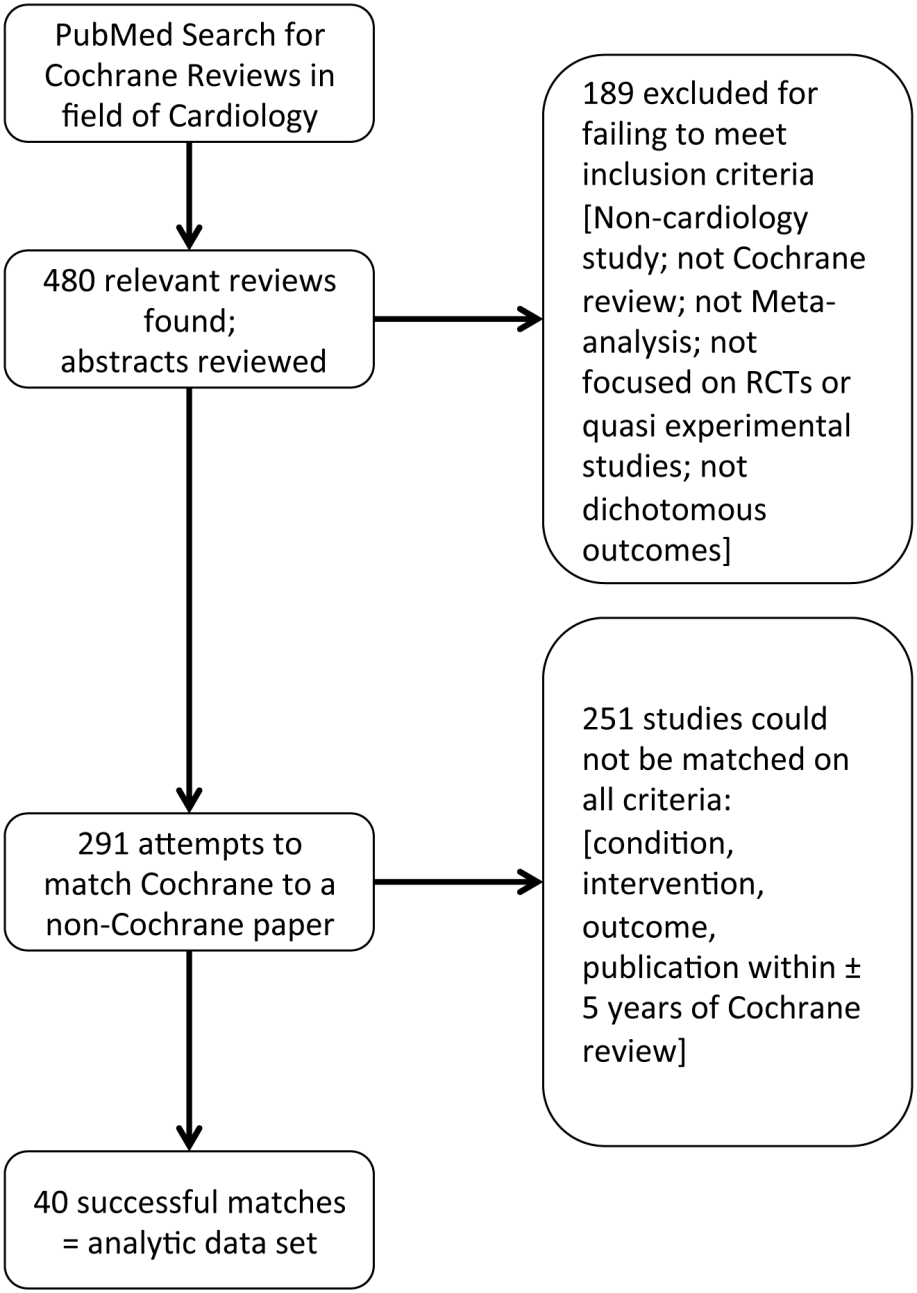
Study selection process and reasons for exclusion of studies. This figure summarizes the path taken from identification of 480 Cochrane meta analyses of randomized controlled trials in the cardiovascular literature through inclusion/exclusion and matching to non-Cochrane reviews to reach our final analytic set of 40 matched pairs of meta-analyses.

The initial search identified 480 Cochrane cardiovascular meta-analyses, of which 189 were excluded based on inclusion/exclusion criteria. From the remaining 291 Cochrane Review articles, we were unable to find a meta-analysis for 251 among the non-Cochrane reviews that matched on disease condition, intervention, outcome measured, and publication within five years. Thus, our search process yielded 40 matched pairs of Cochrane and non-Cochrane meta-analyses that were included in this analysis (Fig 1 and S1 Checklist: PRISMA checklist).

Descriptive statistics of the meta-analyses used in our analyses are shown in Table 1. Cochrane and non-Cochrane reviews were similar in regards to total number of included studies and aggregate sample sizes. The Cochrane meta-analyses included a median of 5.5 (interquartile range (IQR): 3.0–8.5) studies, while non-Cochrane meta-analyses included a median of 6.0 (IQR: 4.5–8.5) studies. The median sample size for Cochrane reviews was 1,368 subjects (IQR: 500–7,788 subjects), while non-Cochrane had a median of 1,434 subjects (IQR: 535–10,485 subjects). The Wilcoxon two-sample test yielded no statistically significant differences between Cochrane and non-Cochrane matches in regards to average sample sizes (p = 0.54) and numbers of studies (p = 0.41) included in each meta-analysis.

**Table 1:** Descriptive features of the 40 matched Cochrane and non-Cochrane meta-analyses.

					Macro differences^1^			Overlap of studies^2^		
Match No.	Lead author (Cochrane is 1st in each pair)	Year	Intervention	Outcome	Publi-cation years*	Sample sizes*	Included studies*	Found in both	Cochrane only	Non-Cochrane only
1	Watson, L. I.	2004	Streptokinase v. UFH	Clot lysis	3	-62	-4	1	2	6
	Wells, Philip S	2001	Streptokinase v. UFH	Clot lysis						
2	Romme, J. J.	2011	Midodrine	Vasovagal syncope recurrence	2	-87	-2	2	0	2
	Liao, Ying	2009	Midodrine	Vasovagal syncope recurrence						
3	Theologou, T.	2011	Preoperative intra aortic balloon pump	In-hospital mortality	3	57	1	2	3	2
	Dyub, Adel M.	2008	Preoperative intra aortic balloon pump	In-hospital mortality						
4	Liakopoulos, O. J.	2012	Preoperative Statin Therapy	Atrial fibrilliation	1	0	0	8	0	0
	Dong, Lili	2011	Preoperative Statin Therapy	Atrial fibrilliation						
5	Martin-Rendon, E.	2008	Stem cells vs. no stem cells	Revascularization	-1	-583	-5	3	2	4
	Zhang, S	2009	Stem cells at 4–7 days vs. none	Revascularization						
6	Crystal, E.	2004	Pacing vs. control	Atrial fibrilliation	-2	-1025	-6	5	3	9
	Burgess, DC	2006	Overdrive/Pacing vs. control	Atrial fibrilliation						
7	Vital, F. M.	2008	CPAP vs. standard care	Mortality	2	82	1	11	1	0
	Peter, JV	2006	CPAP vs. standard care	Mortality						
8	Dieleman, J. M.	2011	Corticosteroids	Atrial fibrilliation	2	343	0	6	11	1
	Marik, Paul E.	2009	Corticosteroids	Atrial fibrilliation						
9	Hofmeyr, G. J.	2010	Calcium supplementation	Gestational hypertension	-1	Indeterminate**				
	Imdad, A	2011	Calcium Supplementation	Gestational hypertension						
10	van Dongen, C. J.	2004	LMWH vs. UFH	Recurrent Venous Thromboembolism	1	Indeterminate**				
	Lorio, A	2003	LMWH vs. UFH	Recurrent Venous Thromboembolism						
11	Clifford, DM	2012	Stem Cells	Mortality	1	964	12	4	14	2
	Tuty Kuswardhani, R.A.	2011	Stem Cells	Mortality						
12	Zhang, S.	2010	Trializad	Symptomatic vasospasm	1	1	0	5	0	0
	Gyoe, Yeon	2009	Trializad	Symptomatic vasospasm						
13	van Dongen, C. J.	2003	Once vs. twice daily LMWH	Recurrent Venous Thromboembolism	2	-227	-2	3	0	2
	Couturaud, F	2001	Once vs. twice daily LMWH	Recurrent Venous Thromboembolism						
14	Taylor, F.	2011	Statins	Mortality	0	-51334	-15	5	3	18
	Tonelli, Marcello	2011	Statins	Mortality						
15	Westendorp, W. F.	2012	Antibiotics	Mortality	3	80	1	4	1	0
	van de Beek, Diederick	2009	Antibiotics	Mortality						
16	Hooper, L.	2004	Omega 3 Fatty Acids vs. placebo/no supplementation	All-Cause Mortality	0	21217	38	6	38	0
	Yezbe, D	2004	Omega 3 Fatty Acids vs. placebo/no supplementation	All-Cause Mortality						
17	Squizzato, A.	2011	Clopidogrel and aspirin	Major cardiovascular events	-1	-10368	-5	2	0	3
	Zouh, YH	2012	Clopidogrel and aspirin	Major cardiovascular events						
18	Fahey, T.	2006	Home-monitoring of blood pressure	Blood pressure control	2	-362	-2	2	0	4
	Cappuccio, FP	2004	Home-monitoring of blood pressure	Blood pressure control						
19	Wright, J. M.	2009	Beta-Blockers	Total Cardiovascular Events	3	8754	1	2	3	2
	Bradley, Hazel A.	2006	Beta-Blockers	Total Cardiovascular Events						
20	De Schryver, E. L.	2003	Dipyridamole plus Aspirin v. Aspirin Alone	Vascular event	-2	-831	7	3	7	2
	Leonardi-Bee, Jo	2005	Dipyridamole plus Aspirin v. Aspirin Alone	Vascular event						
21	Hemmingsen, B.	2011	Intensive glycemic control	Macro vascular complications	1	Indeterminate**				
	Hong, Wu	2010	Intensive glycemic control	Macro vascular events						
22	Akl, E. A.	2007	Anticoagulants	1-year mortality	0	-831	-4	4	1	5
	Kuderer, Nicole M	2007	Anticoagulants	1-year mortality						
23	Lip, G. Y.	2011	Aspirin	Stroke	0	-30740	-7	2	0	7
	Bartolucci, Alfred A.	2011	Aspirin	Stroke						
24	Dong, B.	2006	rt-PA vs. heparin	Major hemorrhage	-3	0	0	5	0	0
	Tardy, B.	2009	rt-PA vs. heparin	Major hemorrhage						
25	Akl, E. A.	2011	LMWH vs.vitamin K antagonists	Mortality	2	226	3	3	3	0
	Louzada, Martha L	2009	LMWH vs. vitamin K antagonists	Mortality						
26	Navaneethan, S. D.	2009	Statin therapy vs. placebo	Cardiovascular mortality	-3	Indeterminate**				
	Palmer, SC	2012	Statin therapy vs. placebo	Cardiovascular mortality						
27	van der Schaaf, I.	2005	Endovascular coiling versus neurosurgical clipping	Cerebral ischemia	0	Indeterminate**				
	de Oliviera, JG	2005	Endovascular coiling versus neurosurgical clipping	Symptomatic Vasospasm						
28	Heran, B. S.	2012	ARBs plus ACEi versus ACEi alone	Mortality	2	-9801	-1	4	3	4
	Kuenzli, Andrea	2010	ARBs plus ACEi versus ACEi alone	Mortality						
29	Nesbitt, C.	2011	Radiofrequency obliteration	Recurrence	3	-15	0	2	1	1
	Luebke, Thomas	2008	Radiofrequency obliteration	Recurrence						
30	Nordmann, AJ	2009	Stenting vs. balloon angioplasty	6–12 month mortality	1	-2956	-6	5	1	5
	De Luca, Giuseppe	2008	Stenting vs. balloon angioplasty	6–12 month mortality						
31	Evers, J. H.	2008	Varicocele surgery or embolisation vs. no treatment	Pregnancy Rate	-3	227	4	3	0	1
	Baazeem, A	2011	Varicocele repair vs. no treatment	Pregnancy Rate						
32	Hoenig, M. R.	2010	Early invasive (angioplasty) vs. Conservative treatment	Index Death or Non-Fatal MI	2	-3793	-4	4	0	4
	O’Donoghue, Michelle	2008	Early invasive (angioplasty) vs. Conservative treatment	Index Death or Non-Fatal MI						
33	Walters, G.	2008	Plasma exchange	Mortality	-3	-134	-4	5	0	4
	Walsh, Michael	2011	Plasma exchange	Mortality						
34	Algra, A.	2006	Oral anticoagulants vs. antiplatelet therapy	Major bleeding complication	-2	-3851	-3	2	1	4
	Schachter, ME	2008	Oral anti-coagulants vs. antiplatelet therapy	Major bleeding complication						
35	Li, W.	2009	Acanthopanax versus control	Improvement of neurological deficit	2	Indeterminate**				
	Wu, B	2007	Traditional Chinese patent medicine	Improvement of neurological deficit						
36	Gabriel, S. R.	2005	Primary and Secondary hormone replacement therapy and placebo	Stroke occurrence	-1	-7931	3	3	6	3
	Magliano, DJ	2006	Hormone therapy vs. placebo	Stroke occurrence						
37	Coward, L. J.	2004	Endovascular treatment vs. carotid endarterectomy	Death or any stroke after 1 year	-3	-334	-1	2	0	1
	Luebke, T	2007	Endovascular treatment vs. carotid endarterectomy	Death or any stroke after 1 year						
38	Rerkasem, K.	2008	Local Anesthesia	MI within 30 days	1	3824	3	4	4	1
	Guay, J.	2007	Local Anesthesia	MI						
39	Brooks, S. C.	2011	Mechanical vs. manual chest compressions	Return of spontaneous circulation	-2	-573	-2	0	2	4
	Westfall, Mark	2013	Mechanical vs. manual chest compressions	Return of spontaneous circulation						
40	Moja, L	2012	Trastuzumab	Congestive heart failure	1	-1601	-2	7	1	3
	Chen, Tao	2011	Trastuzumab	Congestive heart failure						

**Notes**

^1^. ‘Macro differences’ refers the differences in the number of years between the two reviews, the total number of subjects, and the number of studies included.

^2^. The overlap analysis lists the numbers of studies in each pair that were found in both members, or found uniquely in the Cochrane or non-Cochrane reviews.

* Differences are [Cochrane minus (non-Cochrane)]. For example, a Cochrane review published in 2003, with 2000 subjects across 5 studies, and a non-Cochrane published in 2005 with 1000 subjects across 8 studies, would here be summarized as ‘-2’, ‘1000’, and ‘-3’ in the differences in years, subjects and studies categories, respectively.

** In some instances, a review listed the number of articles included, but did not specify whether all of those papers pertained to a specific meta-analysis within the larger report., This occurred primarily in the Cochrane reviews. Since it cannot be assumed that every sources paper was included in every sub-analysis within the overall report, we categorized such instances as ‘indeterminate’ when calculating the macro differences for included studies, sample slizes, and the overlap analysis. The delta publication year of course was unaffected.

Abbreviations:

ACEI: Angiotensin converting enzyme inhibitor; AF: Atrial fibrillation; ARB: Angiotensin receptor blocker; CHF: Congestive heart failure; CPAP: Continuous Positive Airway Pressure; IABP: Intra-aortic balloon pump; INR: International normalized ratio; LMWH: Low molecular weight heparin; MI: Myocardial infarction; PST: Pre-operative statin therapy; rt-PA: Recombinant tissue plasminogen activator; SVT: Supraventricular tachycardia; UFH: Unfractionated heparin; VKA: Vitamin K antagonist; VTE: Venous thromboembolism.

Overall, neither set of reviews dominated in terms of being first to publish. The Cochrane meta-analysis was published before its non-Cochrane pair in 22 of the 40 pairs, while the non-Cochrane meta-analysis was published prior to the Cochrane Review in 13 matched pairs. Five pairs were published in the same year.

The Cochrane and non-Cochrane reviews included a total of 344 individual clinical trials. Of these, 111 (32.3%) studies were included only in a Cochrane review, 104 (30.2%) exclusively in a non-Cochrane review, while 129 (37.5%) had been included in both. Stated another way, 62.5% of studies were only included in one or the other meta-analytic literature, not both. In six instances (Pairs 9, 10, 21, 26, 27 and 35), the overlap between studies included in matched pairs of Cochrane and non-Cochrane reviews could not be determined due to insufficient data within the source paper about which studies had been combined in specific meta-analyses within the larger review (noted as ‘Indeterminate’ in Table 1).

The overlap data in Table 1 allowed us to assess the degree to which publication sequence might account for differences in which studies were included in each member of the matched pair. For example, one might assume that if a non-Cochrane review was published after a Cochrane review and included three studies that the Cochrane did not, that this difference might be explained by studies published subsequent to the first review and only available for the later review. However, we found a large number of matched pairs that violated that assumption. We sorted the 32 matched pairs that were neither indeterminate (Pairs 9, 10, 21, 26, 27 and 35) nor 100% overlapping in their included studies (Pairs 4 and 12) into three groups based on publication sequence. Four pairs were published in the same year (Pairs 14, 16, 22, and 23), of which none had complete overlap. Among the seventeen pairs where the non-Cochrane review was published first, publication sequence alone could not account for the lack of overlap for 8 matches (Pairs 1, 2, 13, 18, 28, 30, 32, and 40). Conversely, among the eleven pairs where the Cochrane review was published first, sequence alone could not account for the lack of overlap for 3 matches (Pairs 20, 31, and 36). Thus, for 47% (15/32) of matched and analyzable non-identical pairs, publication sequence alone would not explain the differences between the lists of included studies between the pairs of Cochrane and non-Cochrane meta-analyses. This suggests that factors other than publication sequence resulted in differences in the inclusion/exclusion of studies in the two sets of reviews.

**Fig 2:**
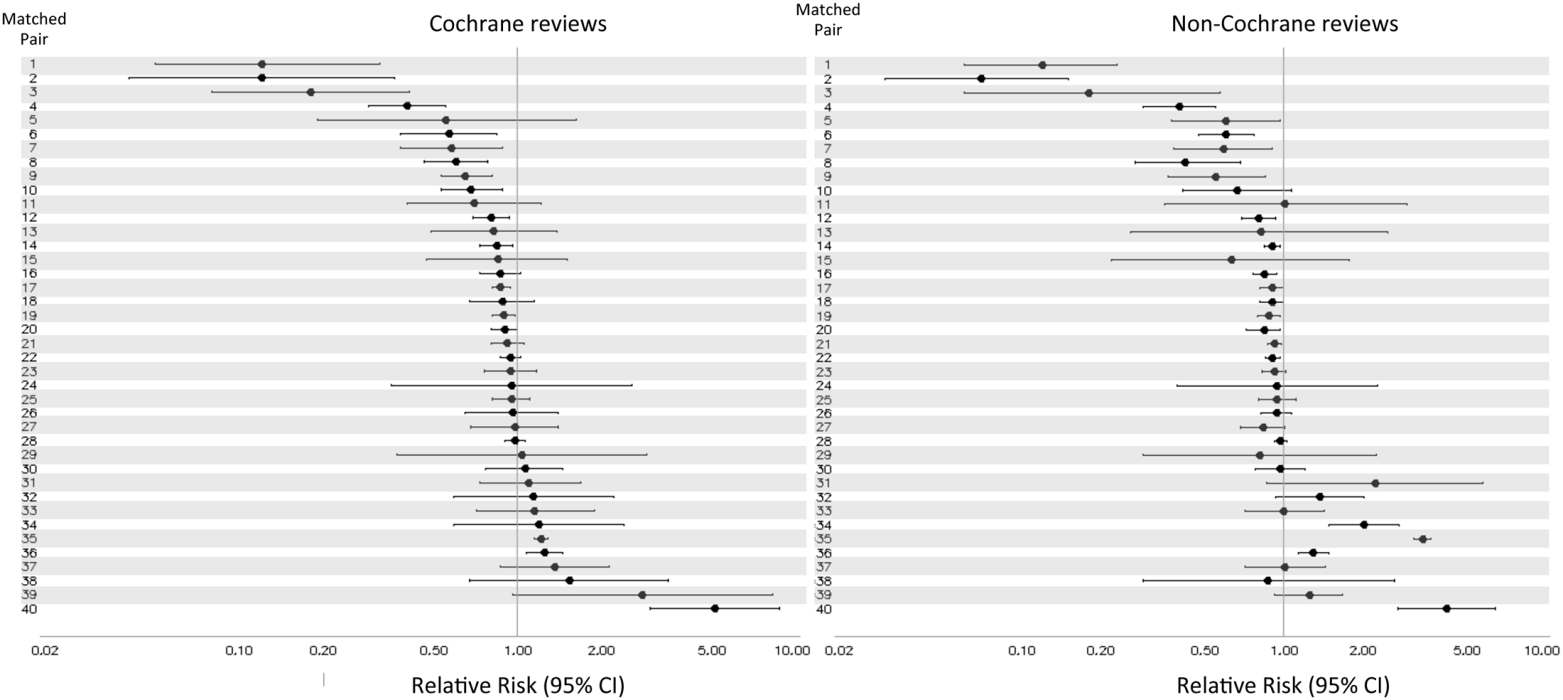
Summary effect sizes for matched Cochrane and non-Cochrane paired meta-analyses. The figure presents Forest plots of effect sizes with 95% confidence intervals for each pair of Cochrane (left) and non-Cochrane (right) reviews. Each of the 40 matched pairs has been sorted based on effect size from the Cochrane review in ascending order of effect size.

Fig 2 provides Forest plots for the reported effect sizes and 95% confidence intervals for each matched pair of reviews going from smallest to largest effect sizes. In all of these analyses, an effect size of 1.0 indicates that the intervention in question had no effect relative to its comparator treatment. The paired analyses were comparable though not identical. As summarized in Table 2, the most frequent discrepancies were shifts in the level of statistical significance due to positioning of the upper or lower bound of the 95% CI relative to 1.0, such that one meta-analysis suggested a statistically significant effect and its match a non-significant effect. This occurred in 7 (17.5%) of the matched pairs. Among the matched meta-analyses that agreed upon the direction of the effect size, 3 (9.1%) differed by at least 2-fold in the magnitude of the effect size. Of more concern, 5 (12.5%) of the matched pairs included a discrepancy such that the direction of the effect size, regardless of statistical significance, either reversed in direction or shifted from a protective to a harmful or null effect, or vice versa. Thus, overall, the results for 15/40 (37.5%) of the paired meta-analyses disagreed to some degree.

**Table 2:** Summary of discrepant results between matched meta-analysis pairs from the Cochrane and non-Cochrane cardiovascular literatures.

Statistical interpretation of the meta analysis changes due to shifts in width of 95% CI around ES (N = 7)	Shifts in the magnitude of ES but in same direction (≥2- fold difference) (N = 3)	Shifts in the direction of the ES (i.e., protective to harmful/null, or vice versa) (N = 5)
Pair 5: Cochrane non-significant; non-Cochrane significant	Pair 31: non-Cochrane ES ≥ 2x Cochrane	Pair 11: Cochrane has NS protective effect; non-Cochrane has NS neutral/harmful effect
Pair 10: Cochrane significant; non-Cochrane non-significant	Pair 35: non-Cochrane ES ≥ 2x Cochrane	Pair 29: Cochrane has NS harmful effect; non-Cochrane has non significant protective effect
Pair 16: Cochrane non-significant; non-Cochrane significant	Pair 39: Cochrane ES ≥ 2x non-Cochrane	Pair 30: Cochrane has NS harmful effect; non-Cochrane has non significant protective effect
Pair 18: Cochrane non-significant; non-Cochrane significant		Pair 33: Cochrane has NS harmful effect; non-Cochrane has neutral effect (~1.0)
Pair 21: Cochrane non-significant; non-Cochrane significant		Pair 37: Cochrane has NS harmful effect; non-Cochrane has neutral effect (~1.0)
Pair 22: Cochrane non-significant; non-Cochrane significant		
Pair 34: Cochrane non-significant; non-Cochrane significant		

**Abbreviations**: CI = Confidence interval; ES = Effect size; NS = non-significant

**Fig 3:**
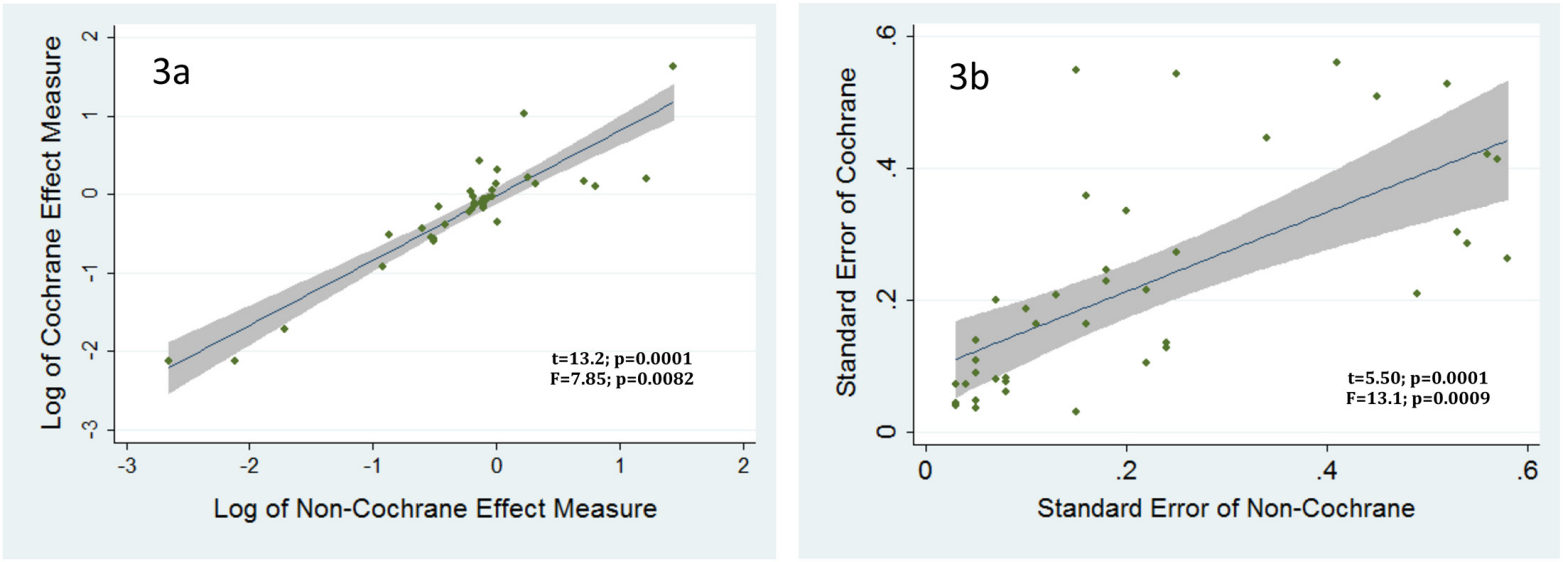
Systematic differences between the Cochrane and non-Cochrane matched meta-analyses, in terms of a) natural log of effect size, and b) standard error of effect size. This figure regresses on a natural log scale pairs of Cochrane and non-Cochrane reviews in terms of effect size (Fig 3a) and standard error (Fig 3b). Each point on the scatter plot represents the intersection point of a Cochrane review with its matched pair in the non-Cochrane literature. In both cases, using T and F tests, the relationships are strongly correlated. However, in both, the slope of the line reveal that, on average, non-Cochrane reviews report slightly larger effect sizes but with larger standard errors (i.e., lower precision) than their matched Cochrane review.

To quantify the degree of concordance between the Cochrane and non-Cochrane literatures, we regressed the summary effect sizes on the logarithmic scale of each pair of reviews (Fig 3a), and their statistical precision using the standard errors of the corresponding effect sizes (Fig 3b). For both outcomes, there were systematic differences separating the two types of reviews. Specifically, non-Cochrane reviews reported significantly larger effect sizes (t = 13.2; p = 0.0001; F = 7.85; p = 0.0082 (Fig 3a)) and wider standard errors (i.e., lower precision around the effect size) (t = 5.50; p = 0.0001; F = 13.1; p = 0.0009 (Fig 3b)) than their matched Cochrane pair.

**Fig 4:**
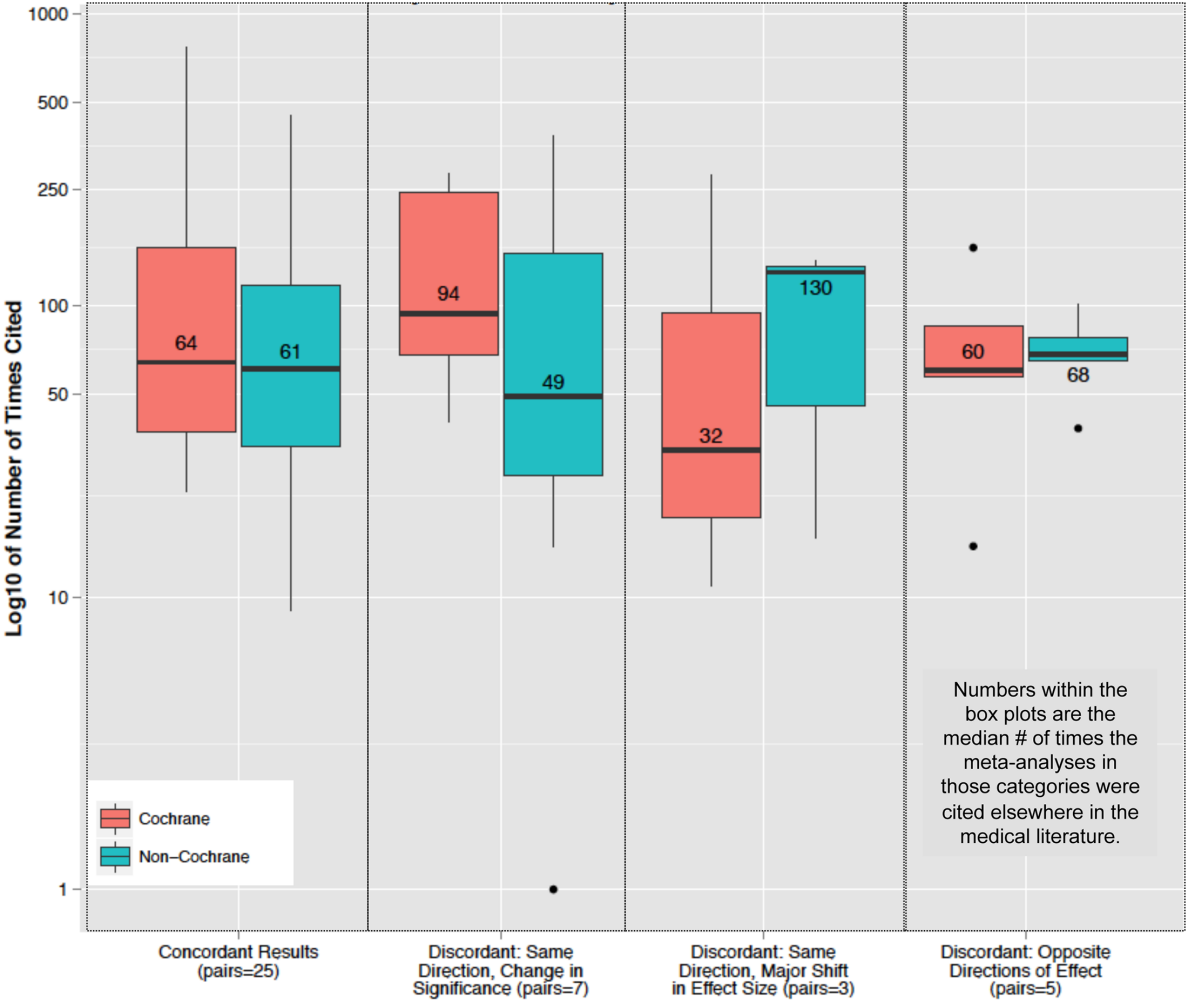
Association between different classifications of discrepant results in matched pairs of meta-analyses and the number of times those analyses were cited elsewhere in the published literature. This figure reports the number of times that Cochrane and non-Cochrane reviews were cited by other articles in the medical literature using the bibliometric feature in Google Scholar. Each pair of box and whisker plots corresponds to a given category of reviews. The first pair lists pairs of reviews that were concordant according to our definitions, meaning that the results of the contrasted analyses agreed. The next three sets of pairs reflect the three different patterns of discrepant results. These were discrepancies based on: shifts in width of confidence intervals that yield a different interpretation of the significant of the effect size (pair 2); instances where one review reported an effect size at least twice that of its match (pair 3); and instances where the effect size reverses (pair 4). To note, the numbers of subsequent citations is quite similar between the Cochrane and non-Cochrane pairs except in the case of discrepancies around the magnitude of the effect size. In those cases, the reviews reporting the larger effect sizes were cited far more often than those reviews reporting the smaller effect size.

We determined the number of times the matched Cochrane and non-Cochrane reviews were cited by other publications as a function of the presence or absence of a discrepant result, and the category of discrepancy when present (Fig 4). Citation rates were very similar between Cochrane/non-Cochrane studies when the results were concordant, if the discrepancy was due to shifts in the 95% confidence intervals, or if the direction of the effect sizes were reversed. However, when the discrepancy was due to a 2-fold or greater difference in the effect size, the reviews that reported the larger of the two effect sizes were cited elsewhere 130 times, vs. 32 times for the matched pair reporting the lower effect size.

## Discussion

The power of meta-analysis lies is its ability to make novel inferences that only emerge when aggregating multiple similar studies on a given topic. By going up one level of aggregation and using individual meta-analyses as the unit of analysis instead of individual studies—in effect a ‘meta-meta’ analysis—we have shown that novel inferences may emerge regarding the meta-analytic literature itself that are only apparent at that higher level of aggregation. Specifically, we have shown that the Cochrane Collaboration’s approach to meta-analysis often yields different results from matched meta-analyses conducted outside of the Collaboration.

While, the matched Cochrane and non-Cochrane meta-analyses were similar in regards to average sample size, the number of individual randomized trials included, and being first to publish, there were frequent differences in the results between the matched Cochrane and non-Cochrane reviews. The most common discrepancy related to shifts in the statistical interpretation of the significance of effect sizes that were otherwise quite similar between Cochrane and non-Cochrane reviews. Such differences are notable given the lamentable tendency of readers to dichotomize results as ‘significant and therefore believable’ vs. ‘non-significant and therefore not believable’.[12] Of more concern, a number of reviews reported markedly different effect sizes, or reported effect sizes that contradicted each other (as from a protective effect to a null or harmful one, or vice versa). These are all instances where the bottom line interpretation by a reader of a review could differ qualitatively.

Moreover, the differences between Cochrane and non-Cochrane reviews appear to be systematic, such that, quantitatively, non-Cochrane reviews report larger effect sizes but with lower precision than matched Cochrane analyses. This supports an earlier analysis by Tricco et al, which, while not a matched pair analysis as in this current study, noted that non-Cochrane reviews were more likely to report positive effects of interventions than Cochrane reviews.[8] Further investigations looking at the quality of included/excluded studies in each literature could help clarify this issue. It was also interesting to note that meta-analyses reporting substantially larger effect sizes than their matched pair were cited roughly 4-times more often in the scientific literature. This is consistent with prior observations that studies or meta-analyses that report larger effect sizes tend to garner more attention in the medical community.[13,14]

Since a meta-analysis is essentially a weighted average of its component studies, it is logical to assume that discrepant results might reflect differences in how studies were selected for inclusion/exclusion by each member of a pair. The generally poor overlap in studies included in the Cochrane and non-Cochrane matched pairs supported this concern. It would be tempting and convenient to explain this simply as one review being published after the other, and thus having access to newer studies that could not have been included in the older paper. However, our results challenged that explanation. First, the two sets of reviews were actually quite well balanced in terms of which was first to publish. More importantly, our data show that publication sequence could only be evoked as a possible explanation for differences in the numbers of included studies in about half of the matched pairs. This argues that these discrepancies are not simply an artifact of time, but more likely reflect differences in search strategies and/or inclusion/exclusion of studies, which may differ systematically between the Cochrane and non-Cochrane literatures.

In other words, our results indicate a substantial divide between the Cochrane and non-Cochrane literatures. What our analysis does not indicate, of course, is which of these sets of reviews is more ‘correct’. With that said, the lower level of precision in effect sizes from the non-Cochrane reviews, combined with lack of standardization of methodology in those reviews, would tend to place the burden of proof on the non-Cochrane side. While our analysis covers a very small fraction of the vast body of work that comprises the meta-analytic literature, readers should be aware that the two types of meta-analyses are not synonymous, and that in some cases the discrepancies could lead to fundamentally different conclusions about whether a given intervention is effective or not.

One of our main limitations is that we only analyzed meta-analyses summarizing data from randomized control trials in the cardiovascular literature, so we cannot comment on whether these results are typical of other medical fields, or even of different kinds of meta-analyses within the cardiovascular field (e.g., of continuous outcomes or diagnostic test precision). Repeating our analysis using meta-analyses from other disciplines could be instructive. We acknowledge that our decision to allow matching of meta-analyses if published within five years of each other could allow for evolutions in the field. However, the difficulty in finding matches at all made it impractical to contract this window. With that said, our overlap analysis suggests that publication sequence was not the most important determinant of which studies were included in a given meta-analysis.

A seeming limitation is that we did not re-analyze the individual studies included in each meta-analysis, but rather took the results from those papers as reported. We defend this decision reasoning that the typical consumer of meta-analytic reports is unlikely to recalculate the results of a paper that he/she is reading, but would also take the reported results at face value. This also applies to the use of different statistical models, such as fixed vs. random effect models. While such differences could lead to shifts in the precision around estimates, it seems exceedingly unlikely that the average reader would go to those lengths to see if recalculating the results would reconcile apparent discrepancies. In other words: our analysis focused only on how the data were presented to the world, not on how they COULD have been presented if using different statistical techniques. Lastly, and most importantly, our analysis offers no insight into which of the two literatures are more likely to provide an unbiased effect size estimate. What we can say is that the results of Cochrane and non-Cochrane reviews frequently disagree, and that the differences appear to be systematic.

The obvious question now is how do we explain these differences? A finding that particularly surprised us in this analysis was that roughly two-thirds of the articles that had been included in the matched reviews were only found in one or the other, but not both. While publication sequence might explain some of this, our overlap analysis showed that this alone could not account for this difference, and therefore the explanation must lie elsewhere. While beyond the scope of this paper, several hypotheses can be suggested: Does one or the other literature tend to miss non-English language publications?; Do they preferentially search different data bases of studies?; Or are the differences related to stricter quality criteria for inclusion? A systematic evaluation of these and potentially other factors is the logical next step for investigation.

In conclusion, this analysis shows yet again how challenging it is to reach a unified interpretation of the medical literature. It is evident that the Cochrane Collaboration’s methodology has many advantages: standardization of methodology, transparency, and the breadth of analyses assessed in one report. Nonetheless, this approach limits the numbers of individuals or organizations that can commit the time and labor to adhering to the Cochrane Collaboration’s standards. One consequence of that is to limit the overall number of analyses that are conducted by the Cochrane Collaboration. Given that meta-analyses are indispensible tools in clinical research, the need for meta-analyses conducted outside of the Cochrane Collaboration is not in dispute. With that said, it is concerning when two meta-analyses addressing the same question, within a similar time frame, reach different conclusions. How should the average doctor or health policy maker react when two ‘gold standards’ disagree with each other? That is an excellent question.

## Supporting Information

S1 Data Final data sets for analysis. (XLSX)

S1 Checklist PRISMA checklist. (DOC)
